# 17*β*-Hydroxysteroid dehydrogenases involved in local oestrogen synthesis have prognostic significance in breast cancer

**DOI:** 10.1038/sj.bjc.6602375

**Published:** 2005-02-01

**Authors:** C Gunnarsson, E Hellqvist, O Stål, and the Southeast Sweden Breast Cancer Group

**Affiliations:** 1 Department of Biomedicine and Surgery, Division of Oncology, Faculty of Health Sciences, Linköping, Sweden

**Keywords:** 17*β*-hydroxysteroid dehydrogenase, breast cancer, oestradiol, real-time PCR, tamoxifen

## Abstract

The 17*β*-hydroxysteroid dehydrogenase (17HSD) enzymes are involved in the local regulation of sex steroids. The 17HSD type 1 enzyme catalyses the interconversion of the weak oestrone (E1) to the more potent oestradiol (E2), whereas 17HSD type 2 catalyses the oxidation of E2 to E1. The aim of this study was to correlate the expression of these enzymes in the tumour with the recurrence-free survival of tamoxifen-treated breast cancer patients. We used real-time reverse transcriptase PCR to investigate the mRNA expression of 17HSD types 1 and 2 in tumour samples from 230 postmenopausal patients. For the patients with oestrogen receptor (ER)-positive breast cancer, we found a statistically significant positive correlation between recurrence-free survival and expression of 17HSD type 2 (*P*=0.026). We examined the ratio of 17HSD types 2 and 1, and ER-positive patients with low ratios showed a significantly higher rate of recurrence than those with higher ratios (*P*=0.0047). ER positive patients with high expression levels of 17HSD type 1 had a significantly higher risk for late relapse (*P*=0.0051). The expression of 17HSD types 1 and 2 in breast cancer differs from the expression of these enzymes in normal mammary gland, and this study indicates that the expression has prognostic significance in breast cancer.

Oestrogens play an important role in the development of hormone-dependent breast cancer. In premenopausal women, the majority of oestrogen is produced in the ovaries. In postmenopausal women, local oestrogens in breast carcinoma tissue originate through two main pathways, one involving aromatase, which converts androgens to oestrogens, and the other utilising steroid sulphatase, which converts oestrone sulphate into oestrone. 17*β*-Hydroxysteroid dehydrogenase (17HSD) activity is finally needed for the oestradiol/ oestrone regulation.

The 17HSD type 1 enzyme uses NADPH as a cofactor and catalyses the interconversion of the weak oestrogen, oestrone (E1), to the biologically more potent oestradiol (E2). 17*β*-Hydroxysteroid dehydrogenase type 2 uses NAD+ as a cofactor and catalyses the oxidation of testosterone and oestradiol to form androstendione and oestrone, respectively (Miettinen *et al*, 1996; Vihko *et al*, 2001). Previous studies have reported the presence of multiple 17HSD isoenzymes in humans, including type 3 and 4 (Speirs *et al*, 1998, 1999; Peltoketo *et al*, 1999), although types 1 and 2 seem to be the principal enzymes involved in reductive and oxidative activity in breast cancer, respectively (Miettinen *et al*, 1999). Moreover, previous studies have suggested that oestradiol can be produced in the same organ where it exerts its biological response. This is in agreement with the fact that breast cancer tissue possesses all the enzymes necessary for the bioformation of oestradiol (Yue *et al*, 1998; Purohit *et al*, 2002).

A few immunohistochemical studies of 17HSD type 1 in human breast cancer have been reported, suggesting that 17HSD type 1 may play an important role in the *in situ* regulation of oestradiol production in hormone-dependent breast carcinomas (Poutanen *et al*, 1992; Sasano *et al*, 1996; Suzuki *et al*, 2000). In a previous study, we found that the expression of both 17HSD types 1 and 2 differ in the tumours of patients with and without late relapse in the disease (Gunnarsson *et al*, 2001).

Oestrogen receptor (ER)-positive breast cancer is usually treated with tamoxifen, and long-term adjuvant tamoxifen is beneficial compared with treatment of shorter duration (Swedish Breast Cancer Cooperative Group, 1996). We hypothesised that the expression levels of 17HSD types 1 and 2, by affecting intratumoural oestradiol levels, might influence the response to endocrine treatment of breast cancer. The purpose of this study was to analyse the prognostic significance of 17HSD types 1 and 2 expression in a series of postmenopausal patients treated with adjuvant tamoxifen.

## MATERIAL AND METHODS

We analysed frozen tissue from excised primary breast tumours of 230 women treated in the health-care region of southeast Sweden between 1985 and 1991. The patients were participants in a randomised multicentric trial where 2 and 5 years of adjuvant postoperative tamoxifen treatment was compared for postmenopausal patients less than 75 years of age (Swedish Breast Cancer Cooperative group). The daily dose of tamoxifen was 40 mg. All patients had primary breast cancer, stage II (UICC), without distant metastasis at the time of diagnosis. The median period of follow-up was 13.9 years. Primary surgery consisted of either modified radical mastectomy or breast-conserving surgery combined with axillary lymph node dissection. Radiotherapy (50 Gy) to the breast was offered to all patients treated with breast-conserving surgery. Lymph node-positive patients were treated with radiation directed to the breast/chest wall and regional lymph nodes. After surgery, the tumour samples were stored in a freezer (−70°C) until RNA extraction was performed. Oestrogen receptor and progesterone receptor (PgR) content was measured in clinical routine practice with isoelectric focusing before 1988 and thereafter with enzyme immunoassays (EIA) (Abbott Laboratories, Chicago, IL, USA). Samples with concentrations ⩾0.1 fmol *μ*g^−1^ DNA (or ⩾0.3 fmol *μ*g^−1^ DNA with EIA) were classified as positive. The present material is a subset of the patients in the region who participated in the trial, and includes the patients for whom frozen tumour samples were available after hormone receptor analysis. The characteristics of the tumours were similar to those in the complete series as regards a positive lymph node status (71 *vs* 74%), tumour size larger than 20 mm (69 *vs* 60%) and a positive ER status (78 *vs* 76%). The study was approved by the regional ethics committee at Linköping University.

### RNA extraction

Frozen breast tumour tissue (30 mg) was homogenised in a microdismembrator (B Braun, Melsungen, Germany), and total RNA was extracted with the SV total RNA isolation system (Promega, Madison, WI, USA). The purified RNA was stored at −70°C, and the RNA content was determined by spectrophotometry. We also examined the expression of 17HSD types 1 and 2 in normal mammary gland. Total RNA from a pool of human breast tissue samples from 3 women (age 46–54 years) was purchased from ADH diagnostics (Life Technologies, Inc.).

### cDNA synthesis

Total RNA (500 ng) was reverse-transcribed in a final volume of 20 *μ*l, using Gibko BRL kit (Life Technologies, Inc., Stockholm, Sweden) with the following concentrations: 1 × PCR buffer, 5 mM MgCl_2_, 0.5 mM deoxynucleotide triphosphates, 2.5 *μ*M random hexamers, 10 mM DTT, and 0.5 *μ*l of Superscript reverse transcriptase (Life Technologies, Inc.) The thermal conditions used were as follows: 20°C for 10 min, 42°C for 50 min, 99°C for 5 min, and after that 5°C. The samples were stored at 4°C as the real-time PCR analysis was performed during the same day.

### Primers and probes

We used the computer software Primer Express (PE Applied Biosystems, Foster City, CA, USA) to design primers and probes that recognised human 17*β*-HSD types 1 and 2 cDNA sequences. We conducted Blast searches (GenBank) to confirm the specificity of nucleotide sequences chosen for the primers and probes and the absence of DNA polymorphism. To avoid detection of contaminating genomic DNA, the probe was placed in the junction between two exons. The primer and probe sequences were; 17HSD type 1: forward primer: 5′-TAT GCG AGA GTC TGG CGG TT-3′, reverse primer: 5′-TGC ACT GGG CCG CAC T-3′, probe: 5′-CGA TCA GGC TCA AGT GGA CCC CAA-3′; 17HSD type 2: forward primer: 5′-TTA CCT GTG GAT CAG AAG GCA GT-3′, reverse primer: 5′-TTG CAC AAA GCA TGG CCA-3′, probe: 5′-CCC GCA ATC ACC ACC TGT CAC CA-3′. Both primers and probes were purchased from PE Applied Biosystems, as were the primers and probes for *β*-actin, which was used as endogenous control gene.

### Real-time PCR

The reactions were performed in the ABI Prism 7700 Sequence Detection System (PE Applied Biosystems). The design of the TaqMan probes, combined with the 5′–3′nuclease activity of AmpliTaq Gold DNA polymerase (PE Applied Biosystems), allows the direct detection of the PCR product by the release of a fluorescent reporter during the PCR.

### PCR conditions

cDNA (3 *μ*l) was added to the reaction mixture, which had a total volume of 25 *μ*l. With the TaqMan PCR core reagent kit (PE Applied Biosystems) the concentrations used were as follows: 1 × TaqMan buffer A, 5.0 mM MgCl_2_, 0.1 mM deoxynucleotide triphosphates, 0.1 *μ*M each of forward and reversed primers, 0.1 *μ*M probe, and 0.025 units *μ*l^−1^ AmpliTaq Gold DNA polymerase. The thermal conditions used were 95°C for 10 min, 95°C for 15 s and 60°C for 1 min. Steps two and three were repeated for 40 cycles. When we used the synthesised cDNA for each tumour, the 17*β*-HSD types 1 and 2 and *β*-actin specific sequences were amplified independently in separate reaction wells in triplicate. On the same plate, we included samples for standard curves for the target genes.

### Standard curve method

A relative kinetic method was applied, using a standard curve, which was constructed with four-fold serial dilutions of cDNA from normal breast tissue. Standard curves were produced for the three target genes after each run. The target messages in unknown samples were quantified, using the standard curves, to determine a relative measure of the starting amount. The measures were normalised, which means that the level of 1 represents the expression level in normal mammary tissue.

### Statistical analysis

The relationships between grouped variables were analysed with the *χ*^2^ test. Survival curves were produced according to the life-table method described by Kaplan and Meier. Differences in recurrence-free survival were estimated with the log-rank test. Multivariate analysis of recurrence and mortality rates was performed with Cox proportional hazard regression. All the procedures are comprised in the statistical package STATISTICA 6.0 (StatSoft Scandinavia AB, Sweden). The criterion for statistical significance was *P*<0.05.

## RESULTS

The 17HSD type 1 enzyme was detected in all of the 230 tumours analysed with a mean value of 2.3. The tenth and the ninetieth percentile for 17*β*-HSD type 1 were 0.1 and 8.2, respectively. To discriminate between low/intermediate and high expression of 17HSD type 1, the material was divided into two groups according to the mean level; lower (<2.3), and higher (>2.3). 17*β*-Hydroxysteroid dehydrogenase type 2 mRNA was detected in 69% of the tumours. The tenth and ninetieth percentile for 17HSD type 2 were 0.0 and 0.92, respectively. The mean expression level for 17HSD type 2 was 0.28. To categorise the patients into two groups the mean value was used as cutoff. Since 17HSD types 1 and 2 cooperate to regulate the levels of E2 and E1, we also determined the ratio between these enzymes in each tumour. The majority showed low ratios as compared to normal. Using the upper tertile as cutoff level, we divided the material into groups with higher (>0.2) or lower (<0.2) ratios. The expression of 17HSD types 1 and 2 were not significantly associated with other tumour characteristics, such as lymph node status, tumour size, ER status or PgR status (Table 1).

### Prognostic value of 17HSD

**Figure 1:**
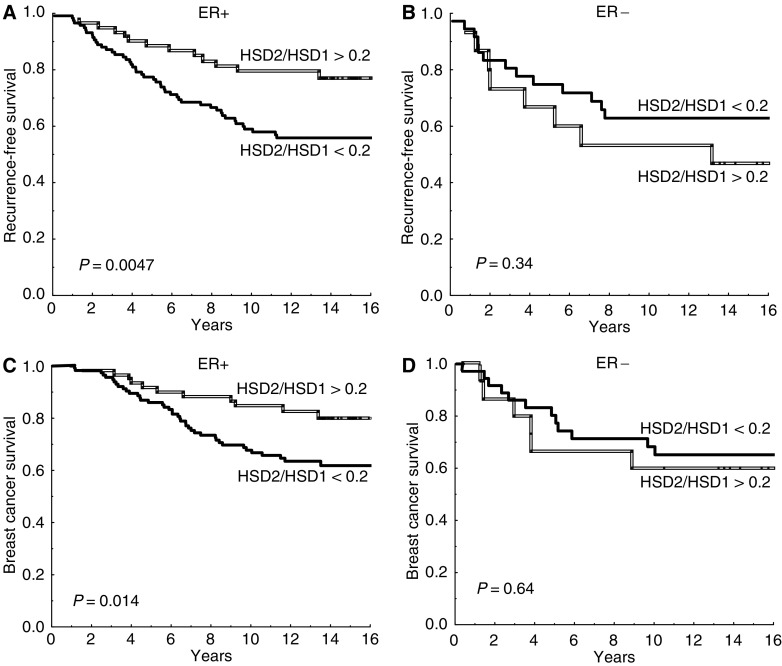
Recurrence-free survival (**A**, **B**) and breast cancer survival (**C**, **D**) in relation to the ratio of 17*β*-hydroxysteroid dehydrogenase (17HSD) type 2 and type 1 mRNA expression (17HSD2/17HSD1). (**A**, **C**) oestrogen receptor-positive (ER+) patients (*n*=179) and (**B**, **D**) ER negative patients (*n*=51).

**Figure 2:**
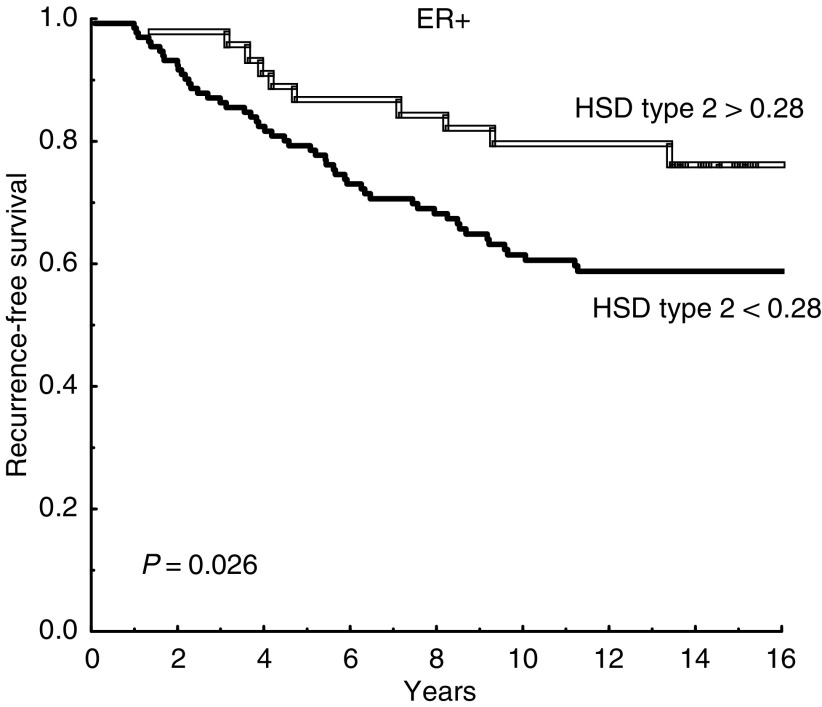
Recurrence-free survival for oestrogen receptor-positive patients with decreased (<0.28) and normal (>0.28) expression of 17*β*-hydroxysteroid dehydrogenase (HSD) type 2 mRNA (*n*=133 and *n*=46, respectively).

**Figure 3:**
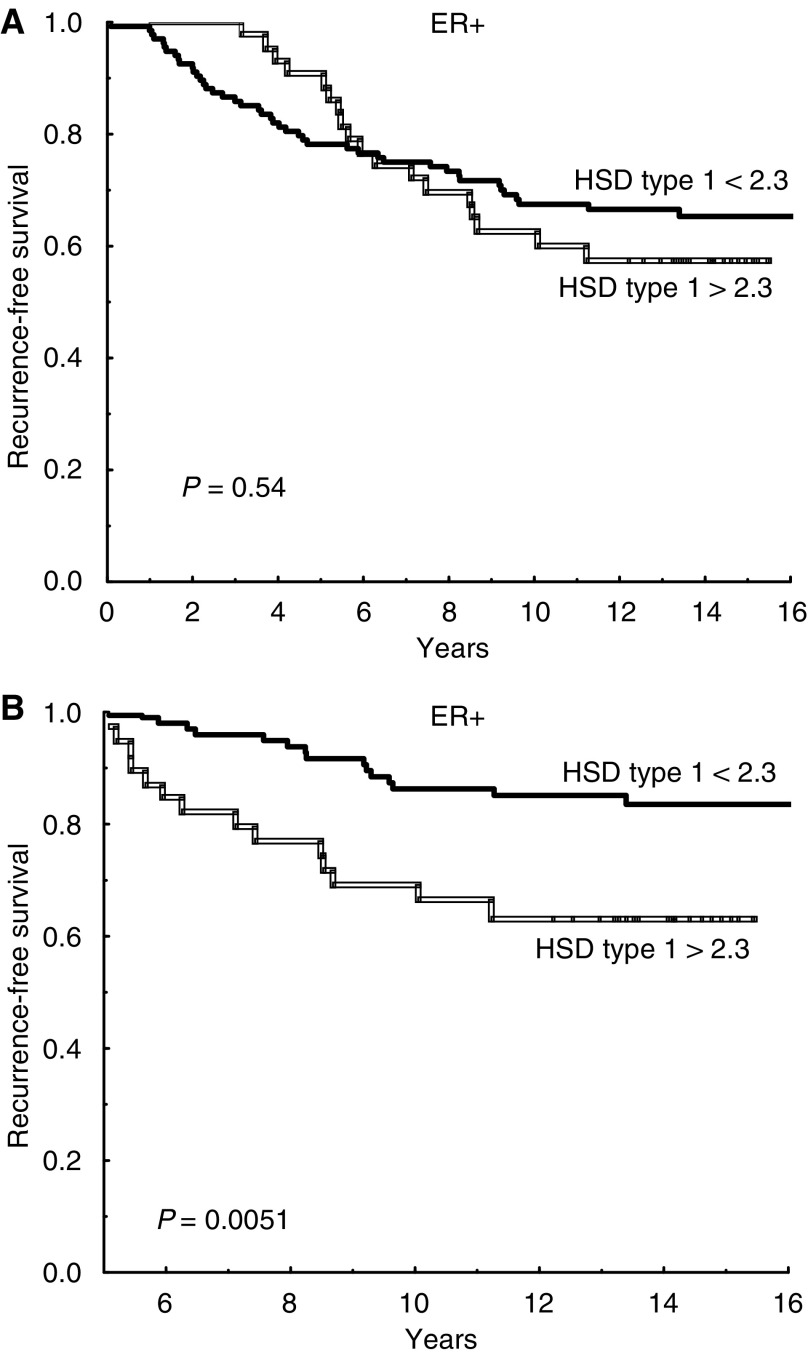
Recurrence-free survival for oestrogen receptor-positive patients with low/normal (<2.3) and increased (>2.3) expression of 17*β*-hydroxysteroid dehydrogenase (HSD) type 1 mRNA (*n*=135 and *n*=44, respectively). The entire follow-up period is shown in (**A**). Patients still recurrence-free after 5 years were analysed for later events and their outcome is shown in (**B**).

Oestrogen receptor-positive patients whose tumour had a high ratio (17HSD2/17HSD1 >0.2) showed a significantly better prognosis than patients with low ratios (*P*=0.0047), whereas no association was found among ER negative patients (*P*=0.34) (Figure 1). This stayed true in multivariate analysis, with regard to recurrence-free survival as well as breast cancer-specific survival (Table 2). A similar result was found for 17HSD type 2 alone. Among ER-positive patients, those with low expression of type 2 had a significantly higher recurrence rate compared with patients who expressed normal levels (*P*=0.026) (Figure 2). This difference could not be seen among ER-negative patients (*P*=0.62). The prognostic significance of type 2 hold true in multivariate analysis (*P*=0.042). There was no significant association between 17HSD type 1 and recurrence-free survival if the entire follow-up period was considered (Figure 3A). However, for ER positive patients still recurrence-free after 5 years, high levels of 17HSD type 1 was associated with a significantly higher rate of late relapse in the disease (*P*=0.0051) (Figure 3B).

### Expression of 17HSD and benefit of 5 *vs* 2 years tamoxifen treatment

Oestrogen receptor-positive patients with low 17HSD2/17HSD1 ratios tended to have the advantage of 5 instead of 2 years tamoxifen treatment (5 *vs* 2 years recurrence rate ratio, RR=0.56, 95% CI, 0.29–1.06, *P*=0.072). This difference could not be seen among patients with high ratios (RR=0.92 (0.28–3.0), *P*=0.90). The same pattern was observed for 17HSD type 2 alone, that is, ER-positive patients with low expression benefited from prolonged treatment, whereas those showing higher type 2 levels appeared to have similar recurrence-free survival with 2 and 5 years of treatment (5 *vs* 2 years, RR=0.60 (95% CI, 0.35–1.04) and RR=1.01 (0.28–3.6), respectively). Oestrogen receptor-positive patients with lower levels of 17HSD type 1 showed a 40% reduced risk of recurrence with prolonged treatment (RR=0.60 (0.33–1.09)), whereas a benefit from prolonged treatment was not evident for those with increased type 1 (RR=1.33 (0.52–3.4)). However, the difference between the groups was not statistically significant.

## DISCUSSION

For ER positive patients in this study, the 17HSD2/17HSD1 ratio had a prognostic significance. A higher 17HSD2/17HSD1 ratio gives an increased oxidative activity (E2 → E1), and in the present study, this was associated with a good prognosis. In contrast, a low ratio leads to increased reductive activity (E1 → E2). Irrespective of E1 production, as a result of aromatase or steroid sulphatase activity, 17HSD types 1 and 2 are responsible for the balance of E1 and E2. It has previously been shown that in normal breast tissue the oxidative pathway (E2 → E1) dominates, whereas in malignant breast tumours the reductive pathway (E1 → E2) is dominant (Speirs *et al*, 1998). These results suggest that intratumoral regulation of oestradiol levels is of importance.

Other enzymes involved in oestrogen synthesis may have prognostic significance, and Utsumi and colleagues (1999) suggested steroid sulphatase as a useful marker for identification of high-risk breast cancer patients. Miyoshi *et al* (2001) observed that intratumoral E2 levels are not significantly different between premenopausal and postmenopausal patients and the authors suggested that upregulation of 17HSD type 1 is important in the maintenance of high intratumoral E2 levels especially in postmenopausal patients. In a more recent study, Miyoshi *et al* (2003) demonstrated that the intratumoral sulphatase mRNA levels, but not the aromatase and 17*β*-HSD1 mRNA levels, have prognostic value in ER-positive breast cancer patients. In the same study, patients with high levels of 17HSD type 1 tended to have a worse prognosis than those with low levels.

The gene encoding 17HSD type 1 is located at 17q12–21, a region that often is rearranged in breast cancer (Plummer *et al*, 1997, Kauraniemi *et al*, 2001). In a recent study, we found amplification of the gene encoding 17HSD type 1 in 14.5% of the breast tumours (Gunnarsson *et al*, 2003).

In the present study, we found that a high expression of 17HSD type 1 predicted late recurrence among ER-positive patients and apparently decreased benefit from prolonged tamoxifen treatment. This could indicate that tamoxifen does not completely block the action of E2 in some patients due to high levels of E2. Acquired tamoxifen resistance is believed to arise due to increased phosphorylation of the ER by growth factor signalling or due to increased expression of coactivators (Nicholson *et al*, 2004, Schiff *et al*, 2004). This means that tamoxifen acts as an agonist after long duration of treatment in some patients, and the question is how the resistant tumour cells will respond to high E2 levels after the completion of tamoxifen treatment. It has previously been shown that tamoxifen treatment for longer periods than 5 years is not preferable (Fisher *et al*, 2001). A recent study pointed out aromatase inhibitor as significantly more efficient than placebo among postmenopausal women after 5 years tamoxifen treatment (Goss *et al*, 2003). Some ER-positive patients with metastatic disease who have failed on tamoxifen treatment still show response to aromatase inhibitors (Dowsett, 1997). In particular, for patients with high intratumoral E2 levels the switch to aromatase inhibitor after some period of tamoxifen treatment could be favourable.

Suzuki *et al* (2000) observed that 17HSD type 1 was immunolocalised in carcinoma cells in a majority of invasive ductal carcinomas, while 17HSD type 2 was not detected in any of the cases. The authors suggested that type 1 is the enzyme of interest in breast cancer. In a previous case/control study, we found that a low or undetectable level of type 2 as well as a high expression of type 1 was associated with a worse prognosis in breast cancer (Gunnarsson *et al*, 2001). The results in the present study are in line with the previous study; however, the present series is larger and not selected as in the case/control study. Among ER positive patients in the present study there was a significant difference in prognosis in relation to 17HSD type 2. Patients who had a low or undetectable level of type 2 had a significantly higher risk of recurrence. These results stayed true in multivariate analysis including other prognostic factors. The loss of 17HSD type 2 expression could result in a significant increase of the more biologically active E2. It has been shown in colon cancer that loss of type 2 is an early event in cancer development, and the authors suggested that type 2 protects the colonic mucosa from E2 (English *et al*, 2001). In a more recent study, Oduwole *et al* suggest that low expression of 17HSD type 2 in colon cancer is an independent marker of favourable prognosis in females. The female patients with high expression of type 2 had a poor prognosis and often tumours with a mucinous component (Oduwole *et al*, 2003).

The gene encoding 17HSD type 2 is located at 16q24 and loss of heterozygosity (LOH) at this site is a frequent and early event in breast cancer (Cleton-Jansen *et al*, 2001). Moreover, in prostate cancer, LOH at 16q is common, and Elo *et al* (1997) discussed whether activity of 17HSD type 2 protects prostatic epithelial cells from excessive androgen action and reduces the proliferative pressure on prostatic cells. Significantly decreased mRNA expression of 17HSD type 2 in prostate cancer as compared to normal prostate tissue has also been reported (Koh *et al*, 2002). In the future, it would be of interest to see if the high frequency of LOH at 16q24 in breast cancer includes the gene coding for 17HSD type 2.

We found that 17HSD type 2 was expressed in the normal mammary gland, whereas the expression levels were low or undetectable in a majority of ER positive breast neoplasms. We also found a favourable prognosis among patients with ER positive tumours that expressed higher mRNA levels of the enzyme. As the loss of 17HSD type 2 activity results in an increased reductive activity (E1 → E2), this might be an important mechanism in the pathogenesis of ER-positive breast cancer. Previous studies have shown most interest in 17HSD type 1. This study indicates that 17HSD type 2 and the 17HSD2/17HSD1 ratio may be even more important, which motivates further investigations.

## Figures and Tables

**Table 1:** 17*β*-Hydroxysteroid dehydrogenase (17HSD) types 1 and 2 expression and the 17HSD2/17HSD1 ratio in relation to tumour characteristics and tamoxifen treatment

	**17HSD1**		**17HSD2**		**HSD2/HSD1**	
	**<2.3**	**>2.3**	**<0.28**	**>0.28**	**<0.2**	**>0.2**
	***n*=171**	***n*=59**	***n*=175**	***n*=55**	***n*=154**	***n*=76**
*Lymphnode status/tumour size (mm)*						
N−, >20	48 (75)	16 (25)	47 (73)	17 (27)	39 (61)	25 (39)
N+, ⩽20	50 (72)	19 (28)	55 (80)	14 (20)	53 (77)	16 (23)
N+, <20	68 (77)	20 (23)	66 (75)	22 (25)	54 (61)	34 (39)

*ER status*						
ER−	36 (71)	15 (29)	42 (82)	9 (18)	36 (71)	15 (29)
ER+	135 (75)	44 (25)	133 (74)	46 (26)	118 (66)	61 (34)

*PgR status*						
PR−	67 (73)	25 (27)	73 (79)	19 (21)	65 (71)	27 (29)
PR+	104 (75)	34 (25)	102 (74)	36 (26)	89 (64)	49 (36)

*Tamoxifen*						
TAM 2 years	74 (64)	41 (36)	81 (70)	34 (30)	76 (66)	39 (34)
TAM 5 years	97 (84)	18 (16)	94 (82)	21 (18)	78 (68)	37 (32)

**Table 2:** Multivariate analysis (Cox) of recurrence rate ratio and breast cancer mortality rate ratio for oestrogen receptor (ER)-positive patients in relation to 17*β*-hydroxysteroid dehydrogenase (17HSD) types 1 and 2 and other variables

	**Recurrence**		**Breast cancer mortality**	
	**RR (95% CI)**	**Significance**	**RR (95% CI)**	**Significance**
*HSD2/HSD1*				
⩽0.2	1.0		1.0	
>0.2	0.43 (0.23–0.80)	*P*=0.0083	0.43 (0.22–0.85)	*P*=0.015

*Nodal status*				
N−	1.0		1.0	
N+	2.2 (1.09–4.3)	*P*=0.027	2.6 (1.2–5.5)	*P*=0.012

*Tumour size (mm)*				
⩽20	1.0		1.0	
>20	1.5 (0.83–2.7)	*P*=0.18	2.1 (1.08–4.2)	*P*=0.030

*PgR status*				
PgR−	1.0		1.0	
PgR+	0.72 (0.42–1.2)	*P*=0.24	0.73 (0.41–1.3)	*P*=0.30

*Tamoxifen*				
2 years	1.0		1.0	
5 years	0.75 (0.45–1.25)	*P*=0.27	0.99 (0.57–1.7)	*P*=0.98
